# 

**Published:** 1882-09

**Authors:** 


Whole Number, CCCCLII.

September, 1882.

Number 3, Vol. XLV.

THE

### CHICAGO MEDICAL Journal and Examiner

EDITORS:

N. S. DAVIS, M.D., LL.D. JAS. NEVINS HYDE, A.M., M.D

DANIEL R. BROWER M.D.

CHICAGO:

*PUBLISHED BY THE CHICAGO MEDICAL PRESS ASSOCIATION,*

188 Clark Street.

☞ Read the Notice of this Journal among Advertisements.

